# Correction: Dependence of the fluorination intercalation of graphene toward high-quality fluorinated graphene formation

**DOI:** 10.1039/d0sc90268c

**Published:** 2020-12-16

**Authors:** Kun Fan, Jiemin Fu, Xikui Liu, Yang Liu, Wenchuan Lai, Xiangyang Liu, Xu Wang

**Affiliations:** College of Polymer Science and Engineering, State Key Laboratory of Polymer Material and Engineering, Sichuan University Chengdu 610065 People’s Republic of China lxy6912@sina.com wangxu@scu.edu.cn +86 28 85405138 +86 28 85403948

## Abstract

Correction for ‘Dependence of the fluorination intercalation of graphene toward high-quality fluorinated graphene formation’ by Kun Fan *et al.*, *Chem. Sci.*, 2019, **10**, 5546–5555, DOI: 10.1039/C9SC00975B.

In the original article, Fig. 5c was displayed incorrectly. A correct version of Fig. 5 is displayed here.

**Fig. 5:**
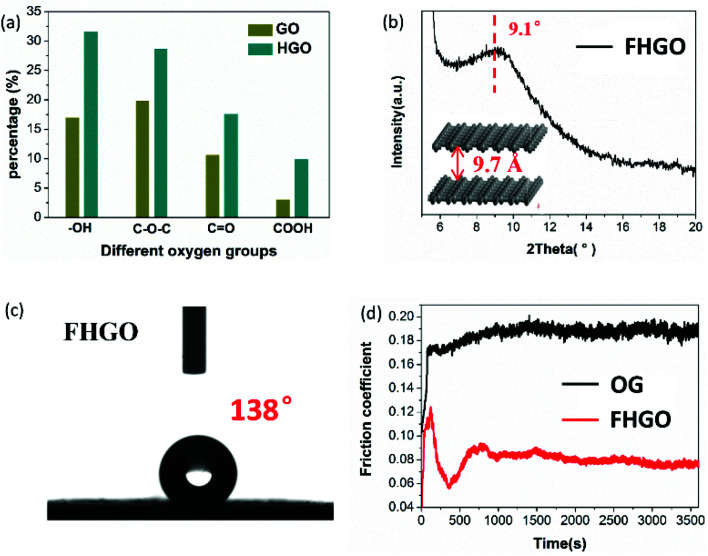
(a) The respective contents of the different oxygen groups (–OH, C–O–C, C=O, COOH) in GO and HGO, (b) the PXRD pattern and the corresponding interlayer distance of FHGO, (c) a photograph of the water contact angle for FHGO, and (d) the friction coefficient lines of OG and FHGO.

Fig. 6 and the description for Fig. 6j were also displayed incorrectly in the original article. The correct version of Fig. 6 and the corresponding description are displayed here.

**Fig. 6:**
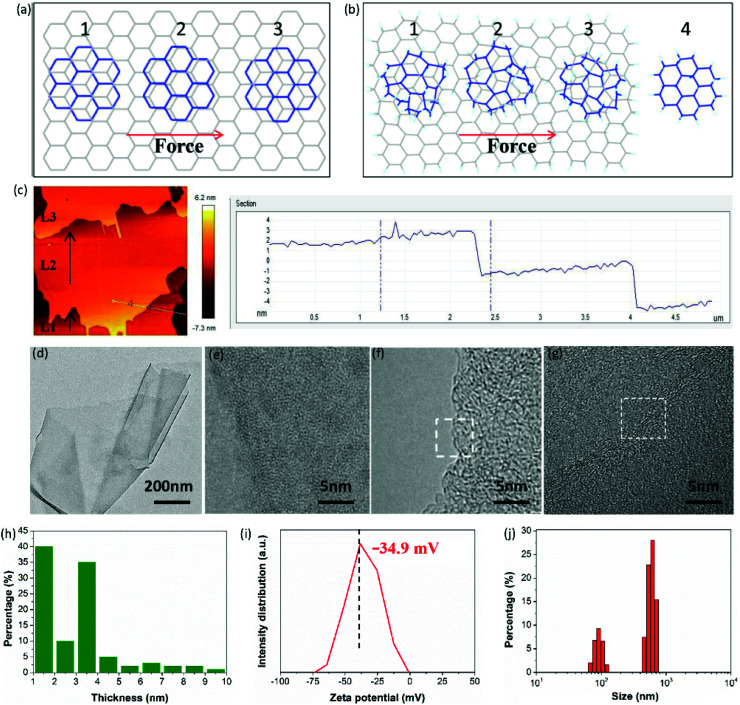
(a) Schematic diagram of a top-graphene sheet moving over a bottom-graphene sheet under shear force, (1) commensurate, (2) incommensurate, and (3) commensurate positions, (b) schematic diagram of a top-FHGO sheet moving over a bottom-FHGO sheet under shear force, (c) AFM picture of FHGO after ball-milling and the corresponding height profile, (d–g) TEM images of exfoliated FHGO at different scales, (h) statistical histogram of the sheet thickness of exfoliated FHGO, (i) zeta potential of exfoliated FHGO, and (j) size distribution of exfoliated FHGO.

On page 5554 of the original manuscript, lines 2–5, the sentence beginning “The size distribution of exfoliated…” should be corrected to read “The size distribution of exfoliated FHGO was characterized by nano-particle size analysis, from 100 nm to 1 mm (Fig. 6j), basically consistent with the results of the AFM measurements (Fig. S16†)”.

On page 5554 of the original manuscript, lines 90–92, the sentence beginning “Dynamic light scattering (DLS) (Nano-ZS, Malvern, UK)…” should be corrected to “Nano-particle size analysis (Nano-ZS, Malvern, UK) was applied to measure the size distribution of the particles”.

These revisions do not alter the scientific conclusions of the manuscript.

The Royal Society of Chemistry apologises for these errors and any consequent inconvenience to authors and readers.

## Supplementary Material

